# Oral Flucloxacillin for Staphylococcal Osteomyelitis: Obsolete or Underused?

**DOI:** 10.7150/jbji.42852

**Published:** 2020-01-01

**Authors:** Staffan Tevell, Bertil Christensson

**Affiliations:** 1 Department of Infectious Diseases, Karlstad Hospital and Centre for Clinical Research, Värmland County Council, Karlstad, Sweden and School of Medical Sciences, Faculty of Medicine and Health, Örebro University, Örebro, Sweden; 2 Department of Infectious Diseases, Skåne University Hospital, Lund and Department of Clinical Sciences, Division of Infection Medicine, Lund University, Lund, Sweden.

## Introduction

In the 1940s, the first published experiences with beta-lactam antibiotics in the treatment of osteomyelitis reported good results, although the examination presented after a short follow-up period possibly overestimated their long-term success. The rationale of antibiotic bone penetration was introduced in the 1970s. This opened the stage for fluoroquinolones and rifampin for the treatment of staphylococcal bone and joint infections 1.

The isoxazolyl penicillins oxacillin, cloxacillin, flucloxacillin and dicloxacillin are semisynthetic penicillins, inhibiting transpeptidase enzymes PBP1a, 1b and 2. Orally administered dicloxacillin and flucloxacillin are absorbed better than oxacillin and cloxacillin, making these the preferred oral agents 2. The clinical value of oral isoxazolyl penicillins for staphylococcal osteoarticular infection and for staphylococcal vertebral osteomyelitis was demonstrated by Hedström *et al.* from the late 1960s onward 3-5 and by Beronius *et al.* in 2001 6. These studies laid the foundation for the Swedish tradition of long-term oral flucloxacillin use in staphylococcal osteomyelitis. The Swedish experience is that even long-term treatment is, in general, well tolerated. Swedish guidelines recommend flucloxacillin as oral follow-up after initial i.v. beta-lactam (1 - 4 weeks) in the treatment of vertebral (3 months) or chronic osteomyelitis without implants (3 - 6 months) in adults 7. In the current issue of the *Journal of Bone and Joint Infection*, Preiss *et al.* 8 present a narrative review on the use of oral flucloxacillin in the treatment of osteomyelitis. The authors conclude that despite concerns about the bioavailability and bone penetration of oral flucloxacillin, the few published case series did not report more clinical failures among patients treated with flucloxacillin than with other oral antibiotic agents. It is, however, difficult to assess whether this result is due to publication bias from authors with positive experiences of this strategy, or due to eradication of remaining bacteria by an unimpaired immune system during long-term oral maintenance therapy. The optimal treatment duration when using beta-lactam antibiotics in osteomyelitis is also unclear, rendering it likely that many patients receive unnecessarily prolonged courses of oral follow-up treatment.

So, in light of recommendations for shorter treatment durations when using fluoroquinolone-rifampin combinations in osteomyelitis in recent years 9, why is it of interest to discuss an agent with wide inter-individual bioavailability, low ratios of bone-to-serum concentrations and prolonged treatment duration?

First, development of resistance during treatment of staphylococcal infections with isoxazolyl penicillins is rare, in contrast to rifampin in which a single point mutation may be enough to lead to resistance 10. As monotherapy with fluoroquinolones is also associated with the development of resistance, combination therapy is crucial for both of these agents. However, resistance rates for fluoroquinolones at baseline among *Staphylococcus aureus* are not negligible, and with recent reports of reduced serum concentrations for several other antibiotic agents when combined with rifampin 11-13, selection of a companion drug for rifampin is difficult in the case of fluoroquinolone resistance or intolerance. Furthermore, in vertebral osteomyelitis, abscesses are common, and prolonged antibiotic therapy is recommended when they are not drained 14. As high bacterial load, inadequate surgical debridement and previous rifampin therapy have been described as risk factors for rifampin resistance 15, rifampin combinations may be inappropriate in vertebral osteomyelitis with an undrained abscess. Thus, to prevent further resistance development, it is crucial that rifampin be used with care and be primarily reserved for indications in which rifampin is necessary for cure (i.e.; as biofilm treatment after debridement, antibiotics and implant retention (DAIR) in implant-associated orthopedic infections) 16.

Second, the tolerability of oral isoxazolyl penicillins is in general good. Skin rash and gastrointestinal side effects are most common, while more severe side effects such as hepatotoxicity and neutropenia are rare 2. Rifampin treatment is plagued by side effects such as nausea, hemolytic anemia, thrombocytopenia, acute kidney failure and hepatotoxicity 17. Regarding fluoroquinolones, both the Pharmacovigilance Risk Assessment Committee (PRAC) of the European Medicines Agency and the U.S. Food and Drug Administration (FDA) have recommended restrictions in their use because of neurological side effects and an increased risk for aortic aneurysms 18-20. Finally, the odds ratios for developing hospital-acquired *Clostridioides difficile* infection are 1.0 (flucloxacillin), 1.6 (fluoroquinolones), 1.7 (trimethoprim-sulfamethoxazole), 1.9 (cephalosporins) and 2.8 (clindamycin) 21. Thus, the use of isoxazolyl penicillins may be beneficial to the patient regarding side effects, even if prolonged treatment duration is required.

In conclusion, oral isoxazolyl penicillins are widely used, with positive experience, for the treatment of osteomyelitis in the absence of implants in some countries, while their use is not endorsed in other countries due to an unfavorable PK/PD profile combined with lack of clinical data. This lack of data is demonstrated with clarity in the narrative review by Preiss *et al*. 8, and it raises the question of whether this has led to overuse of oral isoxazolyl penicillins in countries where this strategy has been traditionally recommended, or an uncalled-for caution in the remaining countries. To approach this question, as is pointed out in the review, those of us treating osteomyelitis patients with oral isoxazolyl penicillins must undertake the challenge of conducting prospective trials, or at least publishing retrospective data on treated patients. Improvement of antibiotic stewardship in osteomyelitis treatment also requires studies that address the question of optimal treatment duration when using isoxazolyl penicillins as oral follow-up. Only through such efforts may we gain more evidence on how to improve care for patients with osteomyelitis.
